# Prolapsus of the Umbilical Cord

**Published:** 1849-02

**Authors:** 


*Prolapsus of the Umbilical Cord*—Is either produced by too great distension of the uterus from liquor amnii, or from the lower portions of the uterus not contracting sufficiently about the child.

Preserve the membranes unruptured as long as possible ; so long as this is the case, the cord is in little danger.

If the passages be well dilated, and the pains active, you may venture to deliver with the forceps ; if not, you must turn the child. Some have succeeded in carrying up the cord upon their hand, and hanging it on some part of the child, and then allowing the head to descend.

Where the cord is without pulsation and flaccid, there will be no need of interfering.—*Dr. Rigby's Obstetric Memoranda.*

